# Inflammatory Markers During Early Treatment of Seroconverters in a Randomized Placebo-Controlled Trial of PrEP (ANRS-IPERGAY)

**DOI:** 10.1093/ofid/ofab085

**Published:** 2021-03-20

**Authors:** Sylvain Chawki, Isabelle Charreau, Audrey Gabassi, Diane Carette, Eric Cua, Laurent Cotte, Gilles Pialoux, Claire Pintado, Laurence Meyer, Marie-Laure Chaix, Constance Delaugerre, Jean-Michel Molina

**Affiliations:** 1 Department of Virology, Hôpital Saint-Louis, Assistance Publique-Hôpitaux de Paris, Paris, France; 2 Maladies Infectieuses, Hôpital Saint-Louis, Assistance Publique Hôpitaux de Paris, Paris, France; 3 INSERM U944, Paris University, Paris, France; 4 INSERM SC10-US19, Villejuif, France; 5 Maladies Infectieuses, Hôpital L’Archet, Nice, France; 6 Maladies Infectieuses, Hospices Civils de Lyon, Hôpital de la Croix-Rousse, Lyon, France; 7 Maladies Infectieuses, Hôpital Tenon, Assistance Publique Hôpitaux de Paris, Paris, France

**Keywords:** CRP, D-dimers, early antiretroviral therapy, HIV, IL6, PrEP, sCD14

## Abstract

HIV-related inflammation is associated with poor outcomes. We describe inflammatory biomarkers in 17 participants in a pre-exposure prophylaxis trial who seroconverted with very early initiation of antiretroviral therapy. Inflammation peaked at the time of HIV infection and returned to baseline within 6–12 months. Starting antiretroviral therapy very early could help mitigate long-lasting HIV-related inflammation.

## Teaser

In seventeen HIV-seroconverters in a PrEP trial who were treated very early on, inflammation peaked at the time of HIV-infection and returned to pre-HIV-infection levels within twelve months. Starting antiretroviral therapy very early could help mitigate long-lasting HIV-related inflammation.

Early initiation of antiretroviral therapy (ART) for the treatment of HIV infection is associated with improved long-term clinical outcome, reduced immune deficiency, limited viral reservoir constitution, and limited HIV transmission [1–3]. Primary HIV infection is associated with marked inflammation (elevated soluble CD14 [sCD14], tumor necrosis factor [TNF]–alpha, interleukin [IL]-6) [1, 4]. It is generally accepted that people treated during symptomatic primary HIV infection have persistent HIV-related inflammation, with inflammatory biomarkers decreasing over time but never returning to normal levels (TNF-alpha, C-reactive protein [CRP], sCD14, and hyaluronic acid) [1, 5]. HIV-related inflammation is associated with poor cardiovascular outcome, metabolic changes, and overall shorter life expectancy, making inflammation the target of innovative therapeutic strategies [6–8].

The efficacy of pre-exposure prophylaxis (PrEP) to prevent HIV infection is particularly established in men who have sex with men (MSM) [9–12]. In case of PrEP failure, HIV-1 diagnosis and ART initiation can occur very early on during the course of infection owing to bimonthly or quarterly repeated screening.

To our knowledge, the evolution of the inflammatory profile of people with HIV compared with their inflammatory profile before the primary infection has not yet been reported.

Here, we longitudinally assessed the inflammation profile of HIV seroconverters participating in a PrEP trial before HIV infection, at the time of primary infection, and during the first year of ART.

## METHODS

### Participants

The ANRS IPERGAY study was a double-blinded randomized trial of PrEP for seronegative highly exposed MSM that took place between February 2012 and June 2016 [11, 12]. The study protocol allowed for sampling plasma, stored at –80°C at each study trial visit: baseline, months 1 and 2, and subsequently every 2 months for all participants. For people with breakthrough HIV infections, blood collection was also performed at the time of HIV diagnosis before starting ART (M0) and 6 months (M6) and 12 months (M12) after ART initiation.

All participants with an HIV diagnosis, by means of plasma HIV-1-RNA or fourth-generation enzyme-linked immunosorbent (EIA-4G) serological assay, were included in this substudy.

### Patient Consent Statement

The study was carried out in accordance with Good Clinical Practices, the ANRS Ethical Chart for Research, and the Declaration of Helsinki. The protocol was approved by national ethics committees in France (Comité de Protection des Personnes de Paris Ile-de-France I). All participants provided written informed consent.

### Inflammatory Biomarkers

Inflammatory biomarkers were measured on plasma when available at the last visit before HIV diagnosis, at the time of HIV diagnosis before starting ART (M0), and at M6 and M12 of ART.

CRP levels were measured with the Cobas third-generation immunoassay (limit of quantification [LOQ] 0.6 mg/L, interassay variability coefficient at LOQ 30%), and IL-6 levels were measured using IL-6 Elecsys Immunoassay kits (LOQ 1.5 pg/mL, variability coefficient 3.1%) with an automated COBAS Modular platform (Roche Diagnostics, Meylan, France). D-dimers were quantified using the LIATEST DdiPlus STA kit (LOQ 270 ng/mL, variability coefficient 7.31%) with a STA-R coagulation analyzer (Stago Diagnostica, Asnières-sur-Seine, France). Human sCD14 (hCD14) was measured with the hCD14 Quantikine ELISA kit (LOQ 250 pg/mL, variability coefficient 7.4%, R&D Systems, Minneapolis, MN, USA). Biomarkers were quantified using consistent reagent lots to eliminate lot-to-lot variability. For CRP, IL-6, and D-dimer, analyses were performed consecutively for each plasma sample after 1 single freeze-thaw cycle. For sCD14, however, all samples were analyzed during 1 single run on the same plate.

### Statistical Analysis

Inflammatory biomarkers were described at each visit as median and interquartile range for continuous variables and as numbers and percentages for qualitative variables and were compared between visits using a Wilcoxon signed rank test. All *P* values and confidence intervals were 2-sided. All analyses were conducted with SAS, version 9.4 (SAS Institute Inc., Cary, NC, USA). All values below the lower limit of quantification were set to 0.

## RESULTS

### Patients

HIV-1 infection occurred in 31 participants during the IPERGAY trial [11, 12]. Plasma samples were available for 17 participants for at least the visit before the diagnosis (n = 17) and 2 other visits among the following: the visit at the time of HIV diagnosis before ART (M0; n = 17), the visit during follow-up at 6 months (M6; n = 14), and/or the visit 12 months (M12; n = 11) after ART initiation. Fourteen patients were in the placebo arm, and 3 were on PrEP [11, 12]. The 3 participants in the PrEP arm were self-reportedly not adherent to the PrEP regimen, and no resistance mutation was detected at HIV diagnosis [11, 12]. All were men with a median age (interquartile range [IQR]) of 34 (27–44) years. Fiebig stages at diagnosis were I (n = 1; 6%), II (n = 7; 41%), III (n = 3; 18%), IV (n = 3; 18%), V (n = 2; 12%), and VI (n = 1; 6%). Ten patients presented with symptomatic primary infection (9 fever and/or flu syndrome, 5 cephalalgia, 2 rashes, and 1 sore throat).

Time between HIV diagnosis and start of ART ranged from 2 to 63 days with a median (IQR) of 6 (3–8) days. Patients were treated with a backbone of TDF and FTC and a third agent including a protease inhibitor in 7 cases (41%); an integrase inhibitor in 8 cases (47%). Two patients (12%) were treated with a combination of 4 drugs: TDF, FTC, and a protease inhibitor associated with an integrase inhibitor in 1 case and a non-nucleoside reverse transcriptase inhibitor in the other. Treatment of HIV infection was at the discretion of the primary care physician.

Immunologic and virologic screening and follow-up test results are presented in Supplementary Table 1. The median viral load (IQR) was initially elevated at 6.3 (4.4–7.0) log_10_ copies/mL and then was suppressed to <20 cp/mL in 75% and 93% of patients after M6 and M12 of treatment, respectively. The median CD4 count (IQR) was 543/mm^3^ (390–770/mm^3^) at the time of diagnosis and increased during ART to 846/mm^3^ (613–975/mm^3^) and 964/mm^3^ (760–1078/mm^3^) at M6 and M12, respectively.

### Inflammatory Biomarkers

**Figure 1.**
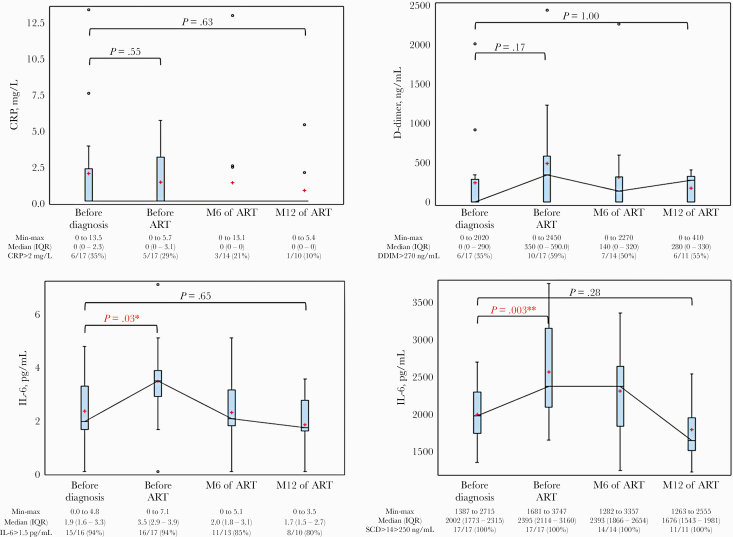
Evolution of inflammatory biomarkers before and after HIV and antiretroviral therapy. The mean for each visit is represented by the red plus sign and the median by the horizontal line across the boxes. All values below the lower limit of quantification were set to 0. The visit “Before diagnosis” happened a median (IQR) of 59 (49–66) days before the following visit. Abbreviations: ART, antiretroviral therapy; CRP, C-reactive protein; DDIM, D-dimer; IL-6, interleukin-6; IQR, interquartile range; sCD14, soluble CD14.

The evolution of biomarkers (CRP, D-dimers, IL-6, and sCD14) is summarized in Figure 1.

At the visit before diagnosis, a median (IQR) of 59 (49–66) days prior, 6 patients had an elevated CRP (defined by a value >2 mg/L), including 3 with a current symptomatic sexually transmitted infection (STI; 1 with gonorrhea, 1 with gonorrhea and chlamydia, and 1 with chlamydia and syphilis). Two patients had elevated D-dimers (>500 ng/mL) at the visit before diagnosis, including 1 with a diagnosis of syphilis and concomitant chlamydia infection. Fifteen patients had elevated IL-6 levels (>1.5 pg/mL), including 8 with a current symptomatic STI (3 chlamydia and 6 gonorrhea, including 1 with both). All patients had elevated sCD14 (>250 ng/mL), including 8 with a current symptomatic STI (3 chlamydia and 6 gonorrhea, including 1 with both).

In the 3 weeks leading up to and after HIV diagnosis, 8 STIs were diagnosed in 5 different participants: 1 oral gonorrhea 1 week before HIV diagnosis, 1 syphilis on the same day as HIV diagnosis, 1 anal chlamydia, 3 anal gonorrhea, and 2 pharyngeal gonorrhea in 3 other participants 4, 5, and 8 days after HIV diagnosis.

The evolution of CRP and D-dimer levels showed no time effect (Figure 1). IL-6 and sCD14 levels were significantly higher at M0 than before HIV diagnosis (*P* = .03 and .003, respectively) and tended to return to baseline levels at M6 and M12. Individual inflammatory profiles are presented in Supplementary Figure 1.

Immuno-virological evolution is presented in Supplementary Table 1.

## DISCUSSION

HIV primary infection led to high levels of inflammation [6]. Even with early initiation of ART, people with symptomatic HIV primary infection tend to have higher chronic levels of inflammatory biomarkers than non-HIV-infected people up to 36 months after ART initiation [1]. Here, we present the results of a study that analyzed the evolution of the inflammatory biomarkers in the setting of very early initiation of ART compared with baseline inflammatory biomarker levels before HIV infection.

We report that in this cohort of high-risk MSM, HIV primary infection was frequently symptomatic and associated with a marked elevation in inflammatory biomarkers. We also noted ~50% elevated inflammatory biomarker levels even before HIV infection. Our study showed that very early treatment of HIV infection (median of 6 days after HIV diagnosis in a cohort with 41% FIEBIG stage II) could allow a return to pre-infection levels of specific inflammatory biomarkers (IL-6 and sCD14) when CRP and D-dimer levels did not seem to change throughout the course of infection.

Our analyses of individual data for inflammatory biomarkers highlight 2 main patterns: (i) elevated baseline inflammation that appears not to be significantly affected by HIV infection followed by a decrease in levels to lower than the individual baseline, hinting toward potential confounders; (ii) inflammation peaking at the time of HIV infection followed by a slow return to baseline levels.

Our study is limited by the small sample size, the retrospective nature of the data, the lack of available CD4 T-cell counts at the time point before HIV infection, and a high proportion of participants with CRP or D-dimer levels below the limit of quantification. Five participants were diagnosed with STIs around the time of HIV diagnosis; this should not affect the overall results of the inflammatory biomarkers presented here due to the wide temporal spread of these infections from HIV diagnosis and the relatively small number of events. The test/*P* value for each biomarker at baseline was compared with the time of HIV diagnosis and month 12 and was not adjusted for multiple comparisons because of the limited number of observations. This analysis was exploratory and centered on a controlled and carefully selected number of conditions. Additionally, due to significant missing data at month 12, bars and raw medians between baseline and month 12 should not be compared in Figure 1.

Measuring ultrasensitive CRP levels might increase sensitivity and uncover fluctuating inflammation for all participants with a value <2 mg/L.

Overall, in a context of elevated HIV-related inflammation, very early treatment leads to a rapid decrease in inflammatory biomarkers and might allow a return to pre-infection levels. This decrease might in turn lead to better long-term outcomes. Our study reinforces the recommendation of early treatment for people who seroconvert while taking PrEP. Future larger studies should consider prospectively monitoring inflammatory biomarkers in people who seroconvert while on PrEP and in those with STIs to confirm these results.

## Supplementary Data

Supplementary materials are available at Open Forum Infectious Diseases online. Consisting of data provided by the authors to benefit the reader, the posted materials are not copyedited and are the sole responsibility of the authors, so questions or comments should be addressed to the corresponding author.

ofab085_suppl_Supplementary_Figure_S1

ofab085_suppl_Supplementary_Table_S1
