# Supplementary figures and images for: Inflammatory Markers During Early Treatment of Seroconverters in a Randomized Placebo-Controlled Trial of PrEP (ANRS-IPERGAY)
Source: Open Forum Infect Dis. 2021 Mar 20;8(3):ofab085. doi: 10.1093/ofid/ofab085 (PMC7990514; doi:10.1093/ofid/ofab085)

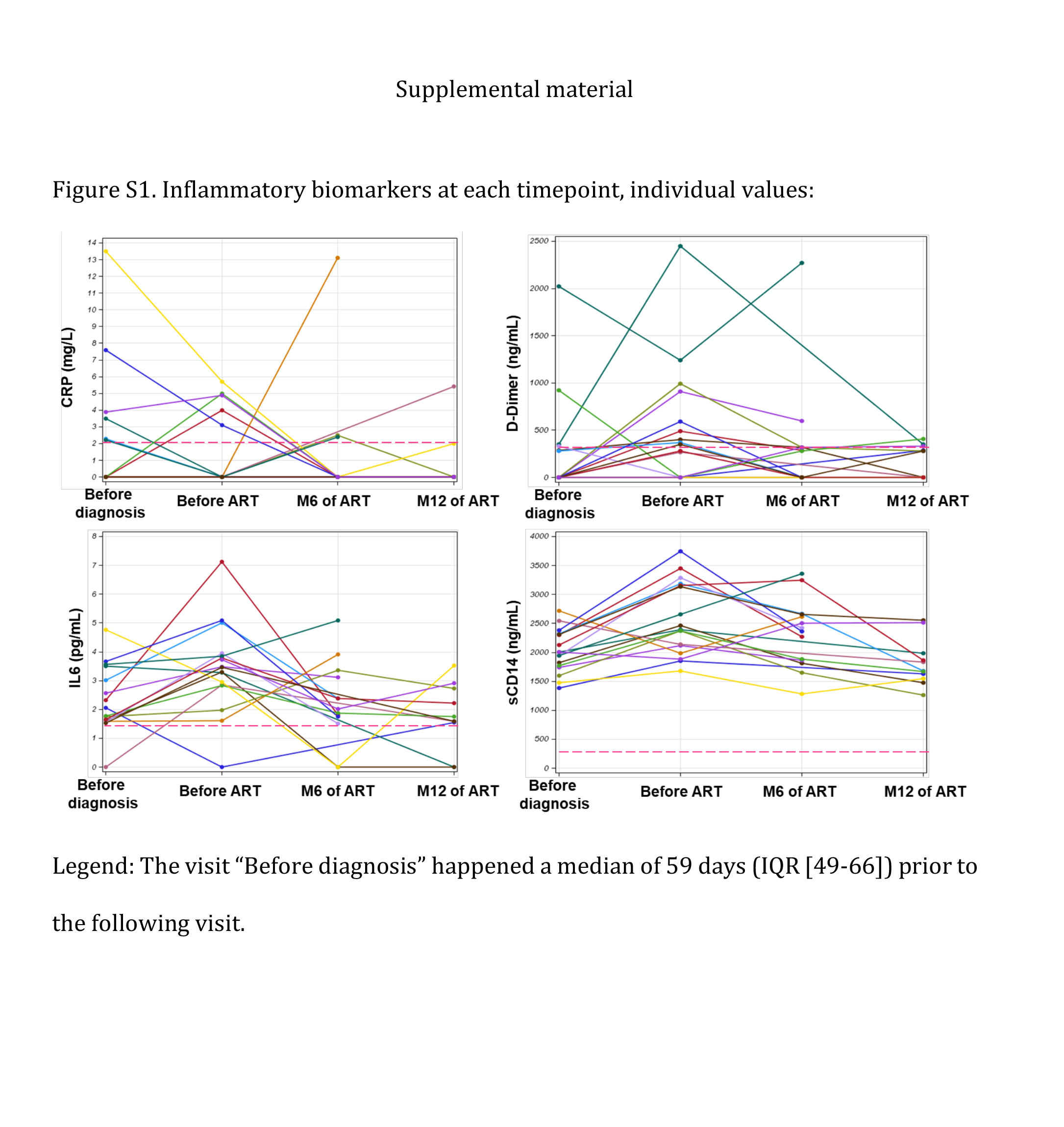

Supplement: ofab085_suppl_Supplementary_Figure_S1 [file ofab085_suppl_supplementary_figure_s1.png]
