# Supplementary material for: Inflammatory Markers During Early Treatment of Seroconverters in a Randomized Placebo-Controlled Trial of PrEP (ANRS-IPERGAY)
Source: Open Forum Infect Dis. 2021 Mar 20;8(3):ofab085. doi: 10.1093/ofid/ofab085 (PMC7990514; doi:10.1093/ofid/ofab085)
Supplement: ofab085_suppl_Supplementary_Table_S1 [file ofab085_suppl_supplementary_table_s1.docx]

Supplemental material

Table S1 Immunologic and virologic marker of HIV infection at each visit

| **Criteria** | **M0**  **(after diagnosis before ART)**  **n=17** | **M6 of ART**  **n=16** | **M12 of ART  n=14** |
| --- | --- | --- | --- |
|  | **n/N total (%) or median [IQR]** | **n/N total (%) or median [IQR]** | **n/N total (%) or median [IQR]** |
|  |  |  |  |
| **Diagnostic tests** | | | |
| Western blot (number of antibody) | 3 [0;7] | 8 [7;9] | 7 [6;9] |
| Positive Home test | 8/16 (50) | 12/12 (100) | 8/11 (73) |
| Positive ALERE p24 **Antigen** | 5/16 (31) | 0/12 (0) | 0/11 (0) |
| Positive ALERE p24 **Antibody** | 12/16 (75) | 12/12 (100) | 11/11 (100) |
| **Immunovirological outcome** | | | |
| CD4 count /mm3 | 543 [390;770] | 846 [613;975] | 964 [760;1078] |
| Plasma HIV RNA |  |  |  |
| <20 copies/ml | 0 (0) | 12/16 (75) | 13/14 (93) |
| log_10_ copies/ml | 6.3 [4.4;7.0] | 1.6 [1.3;1.7] | 1.5 [1.3;1.6] |
| Cell-associated HIV DNA |  |  |  |
| < 40 copies/millions CD4 | 1/16 (6) | 4/14 (29) | 2/10 (20) |
| log_10_ copies/millions CD4 | 4.0 [3.3; 4.5] | 2.5 [0.0; 3.1] | 2.7 [0.1; 3.0] |
